# Artificial selection causes significant linkage disequilibrium among multiple unlinked genes in Australian wheat

**DOI:** 10.1111/eva.12807

**Published:** 2019-07-18

**Authors:** Reem Joukhadar, Hans D. Daetwyler, Anthony R. Gendall, Matthew J. Hayden

**Affiliations:** ^1^ Department of Animal, Plant and Soil Sciences La Trobe University Bundoora Victoria Australia; ^2^ Agriculture Victoria Research, AgriBio Centre for Agribioscience Bundoora Victoria Australia; ^3^ School of Applied Systems Biology La Trobe University Bundoora Victoria Australia

**Keywords:** australian wheat, flour quality, grain yield, selective sweeps

## Abstract

Australia has one of the oldest modern wheat breeding programs worldwide although the crop was first introduced to the country in 1788. Breeders selected wheat with high adaptation to different Australian climates, while ensuring satisfactory yield and quality. This artificial selection left distinct genomic signatures that can be used to retrospectively understand breeding targets, and to detect economically important alleles. To study the effect of artificial selection on modern cultivars and cultivars released in different Australian states, we genotyped 482 Australian cultivars representing the history of wheat breeding in Australia since 1840. Computer simulation showed that 86 genomic regions were significantly affected by artificial selection. Characterization of 18 major genes known to affect wheat adaptation, yield, and quality revealed that many were affected by artificial selection and contained within regions under selection. Similarly, many reported QTL and genes for yield, quality, and adaptation were also contained in regions affected by artificial selection. These included *TaCwi‐A1, TaGw2‐6A, Sus‐2B, TaSus1‐7A, TaSAP1‐7A, Glu‐A1, Glu‐B1, Glu‐B3, PinA, PinB, Ppo‐D1, Psy‐A1, Psy‐A2, Rht‐A1, Rht‐B1, Ppd‐D1, Vrn‐A1, Vrn‐B1*, and *Cre8*. Interestingly, 17 regions affected by artificial selection were in moderate‐to‐high linkage disequilibrium with each other with an average *r* ^2^ value of 0.35 indicating strong simultaneous selection on specific alleles. These regions included *Glu‐B1, TaGw2‐6A, Cre8, Ppd‐D1, Rht‐B1, Vrn‐B1, TaSus1‐7A, TaSAP1‐7A*, and *Psy‐A1* plus multiple QTL affecting wheat yield and yield components. These results highlighted the effects of the long‐term artificial selection on Australian wheat germplasm and identified putative regions underlying important traits in wheat.

## 1 INTRODUCTION

Bread wheat (*Triticum aestivum*; 2n = 6x = 42; AABBDD) evolved around 10,000 years ago (Dubcovsky & Dvorak, 2007) through a spontaneous interspecific hybridization between *Aegilops tauschii* (2n = 2x = 14; DD) and tetraploid emmer wheat *Triticum turgidum* (2n = 4x = 28; AABB) and has been domesticated by humans ever since. The history of bread wheat in Australia is relatively short, as it was first introduced to the country by the European first fleet settlers in 1788 (Henzell, 2007). The history of Australian wheat can therefore be best described as 230 years of crossing and artificial selection. The first few decades were not very successful for Australian wheat growers as they used European (predominantly British) cultivars which evolved in completely different climates and latitudes, and consequently, Australia relied on the importation of a considerable proportion of its wheat demand (Henzell, 2007). Since then, Australian wheat germplasm had two major shifts (Pugsley, 1983). The first was at the beginning of the 20th century when William James Farrer developed the early‐maturing and high‐quality cultivar “Federation,” through successful cross‐breeding between three cultivars of broad geographical origins (Henzell, 2007). The second has occurred since the beginning of the Green Revolution in 1970, where cultivars developed at the International Maize and Wheat Improvement Center (CIMMYT) have dominated breeding efforts across the country (Brennan & Quade, 2006; Joukhadar, Daetwyler, Bansal, Gendall, & Hayden, 2017). Previous studies have shown that cultivars released before and after 1900 are genetically similar but differentiated from those released after 1970 (Joukhadar et al., 2017). Today, Australia is one of the largest wheat exporters globally, due to the extensive efforts of the Australian wheat breeders in crossing and selecting high‐yielding and high‐quality cultivars adapted to Australian climates.

Although breeding efforts have improved the genetic gain for different traits in Australian wheat, the overall genetic diversity in modern cultivars is reduced when compared to older cultivars (Joukhadar et al., 2017). This reduction is a result of repeatedly selecting for the same desirable alleles that are controlling economically important traits from a limited gene pool. For example, the semidwarf alleles *Rht‐B1b* and *Rht‐D1b* became the main target for selection after the Green Revolution (Rebetzke & Richards, 2000). Different allele combinations for the photoperiod (*Ppd*) and vernalization (*Vrn*) genes that control flowering time have been selected for wheat grown in different agriproduction zones across Australia (Cane et al., 2013; Eagles et al., 2010; Eagles, Cane, & Vallance, 2009). Multiple quality genes have been also subject to selection across Australia depending on industrial end‐use requirements (Cane et al., 2008; Crawford et al., 2011; Eagles et al., 2006). The cereal cyst nematode (CCN) resistance gene *Cre8* has been widely adopted across Australia (Jayatilake et al., 2015; Safari et al., 2005; Williams, Willsmore, Olson, Matic, & Kuchel, 2006).

Both artificial and natural selection cause detectable changes in the allele frequencies of the selected sites and their flanking regions. Several statistical methods have been developed to detect selection signatures in natural populations, and their concepts fall into one of three main categories: first, methods that detect abnormalities in the allele frequency spectrum within a single population such as the composite likelihood ratio (CLR) method (Nielsen et al., 2005); second, methods that target distortion in linkage disequilibrium or haplotype structure in a single population such as the integrated haplotype score (iHS) or the number of segregating sites by length (nSL) method (Ferrer‐Admetlla, Liang, Korneliussen, & Nielsen, 2014; Voight, Kudaravalli, Wen, & Pritchard, 2006); and third, methods that exploit differentiation between populations such as *F*_st_ (Weir, 1996) and the cross‐population composite likelihood ratio test (XPCLR; Chen, Patterson, & Reich, 2010). Artificial selection can be also detected using methods proposed to identify natural selection (Beissinger et al., 2014). However, detecting selection in breeding populations has two advantages over that in natural populations. Firstly, the founding parents of breeding populations are usually available, which allows a better estimation of allele frequency changes, compared to natural populations that encounter long‐term simultaneous selection across a large number of traits (Beissinger et al., 2014). Secondly, experimental populations are typically much younger than natural populations, which increases the detection power particularly for haplotype‐based methods due to the smaller number of historical recombination events (Tang, Thornton, & Stoneking, 2007).

Differentiating true signals of selective sweep from arbitrary genetic drift is the major problem affecting the power of different population genetics studies such as genomewide selection scans. Irrespective of the statistical method, the approach widely used to declare a locus to be under selection, that is, when its score is an outlier given the empirical distribution of the statistical test, has been shown to reduce the detection power and to cause very high false‐positive or false‐negative error rates (Kelley, Madeoy, Calhoun, Swanson, & Akey, 2006; Teshima, Coop, & Przeworski, 2006). The problem was reported to be best addressed by simulating the empirical population under a neutrality assumption to compare the observed allele frequencies with those expected under genetic drift only. This approach enables significance thresholds specific to the studied population to be set (Pavlidis & Alachiotis, 2017). Similarly, empirical factors that can potentially have confounding effects on the results, such as ascertainment bias associated with SNP chip data, can also be simulated to take their distorting effect into consideration (Albrechtsen, Nielsen, & Nielsen, 2010).

In this study, we used 482 cultivars representing the history of wheat breeding in Australia since 1840 to scan for signatures of artificial selection. We also characterized the germplasm for allelic variation at 18 genes that control important traits. We compared post‐Green Revolution (Post70) with pre‐Green Revolution (Pre70) varieties, as well as cultivars released in different Australian states for both artificial selection tests and characterized genes. We attempted to identify the specific traits underlying the selective sweeps by examining their association with characterized genes and published QTL known to affect wheat adaptation, yield, and quality traits.

## 2 MATERIALS AND METHODS

### 2.1 Plant materials and genotyping

The 482 Australian cultivars used in this study were released between 1840 and 2011. Seed and pedigree information was obtained from the Australian Grains Genebank, Horsham, Victoria. The germplasm was genotyped using an Infinium iSelect 90K SNP bead chip assay (Wang et al., 2014). Full experimental details and genotype information were previously described in Joukhadar et al. (2017). Cultivars released after 1970 were considered the post‐Green Revolution cultivars (Post70).

### 2.2 Amplicon resequencing

Eighteen genes known to affect wheat adaptation, yield, and grain quality traits that could have been targets of selection in Australian wheat breeding program were selected for amplicon resequencing: the characterized genes that controlled agronomic traits selected for analysis: *Sus‐2B* (sucrose synthase 2)*, Ppd‐D1* (photoperiod)*, Rht‐B1, Rht‐D1 *(plant height)*, Vrn‐A1, Vrn‐B1*, and *Vrn‐D1* (vernalization); those affecting quality traits: *Glu‐A1, Glu‐B1, Glu‐B3, Glu‐D1 *(glutenin)*, Ppo‐A1, Ppo‐D1 *(polyphenol oxidase)*, Wx‐B1 *(waxy)*, PinA, PinB* (puroindoline), and *Psy‐A1* (phytoene synthase 1); and the CCN resistance gene *Cre8*. A total of 31 PCR primers were used to characterize major alleles for these 18 genes (Table S1). PCR primers targeted the variants that differentiate the targeted alleles as described in Table S1. PCR primers were designed using Primer3 and were aligned to the wheat reference genome using *GYDLE* software (https://www.gydle.com/) to ensure that they would not amplify multiple copies. All PCR products for each sample were quantified using qPCR, and the PCR products were pooled together. The pooled amplicons were cleaned using SPRI magnetic beads with a ratio of DNA:beads of 1:2. DNA libraries were prepared for next‐generation sequencing using the KAPA Hyper Prep Kit. After attaching a unique barcode for each sample, samples were pooled in two groups and cleaned using SPRI magnetic beads with a ratio of DNA:beads of 1:1. A KAPA Library Quantification Kit was used to quantify the library for sequencing. The pooled amplicons (two pools) were sequenced using two runs on an Illumina MiSeq (Reagent Kit v3, 0‐ to 300‐nt paired‐end reads). The runs generated 30.03 million reads with number of reads per sample for all amplicons ranging from 21,914 to 364,269.

### 2.3 Analysis of resequencing data

The raw FASTQ files were first processed with the *fastq_quality_trimmer* from the FASTX toolkit (http://hannonlab.cshl.edu/fastx_toolkit/). The minimum quality threshold was set to 37, and reads shorter than 150 nt were discarded. Paired reads were then aligned to the IWGSC genome assembly v0.3 of wheat cultivar Chinese Spring (https://www.wheatgenome.org) using *GYDLE* software (https://www.gydle.com/) to ensure they were derived from the targeted locus. The amplicons were then divided into two classes. Class I amplicons exhibited SNP variants or short insertions–deletions. Class II amplicons contained large insertions–deletions or multiple variants in which many alternative alleles could not be aligned unambiguously to the reference allele. The MEM algorithm implemented in the *BWA* aligner (Li, 2013) was used to align the high‐quality paired‐end reads to the reference with a minimum seed length equal to 30 or 100 for the Class I amplicons and Class II amplicons, respectively. The resulting SAM files were processed with *SAMtools* (Li et al., 2009). Alignments with MAPQ values smaller than 10 were discarded, and only properly aligned pairs were kept. The Class I amplicon paired‐end reads were aligned to the reference allele, and *SAMtools* was used to generate an mpileup file. Variants were called in VCF format with the *VarScan* software (Koboldt et al., 2012) using the mpileup2cns option with a minimum of 10 reads and threshold p‐value of 0.01. The Class II amplicon paired‐end reads were aligned to a reference that included all expected alleles for each amplicon, and an extra filtering step was performed in which only paired reads that exactly matched and had the same insert size as one of the reference alleles were kept. This is appropriate for this study as the majority of cultivars used in this study are expected to have one of these known alleles, and because rare or novel alleles were not important for the purpose of this study.

### 2.4 Genomewide scan for selection signatures

Four different statistical approaches were used to detect selection signals: *F*_st_, XPCLR, iHS, and nSL. *F*_st_ was calculated following Nicholson et al. (2002). *F*_st_ values were averaged using a sliding window size of 15 SNPs, with a step size of one SNP. The window size was chosen considering the linkage disequilibrium and marker coverage in this germplasm, as previously described (Joukhadar et al., 2017). The XPCLR (Chen et al., 2010) scan was run with a 1 Mb grid size and 1 cM window size. The maximum number of SNPs within each window was set to 50. One random SNP was selected for each pair of SNPs with *r^2 ^*> 0.95 within each window. The default parameters for iHS and nSL were used in our analysis as described in Voight et al. (2006) and Ferrer‐Admetlla et al. (2014). For all analyses, adjacent windows that passed the significance threshold and showed strong LD were considered as a single signature. The single‐population methods iHS and nSL were also applied to the whole population with 482 cultivars. SNP genetic positions were obtained from Wang et al. (2014). Detected regions were compared to previously reported QTL in Australian or CIMMYT germplasm to define potential candidate genes or QTL subject to selection within these regions. Genetic positions were compared across different previously published maps using the *CMap* tool (http://ccg.murdoch.edu.au/cmap/ccg-live/).

### 2.5 Simulating the diversity of Australian wheat germplasm

The polyploid genome simulator (PolySim) was used to simulate the Australian hexaploid wheat germplasm (Jighly et al., 2018). One hundred replicates with different random seeds for the following simulation scheme were conducted. Each replicate started with 500 individuals of a common ancestor population with seven diploid chromosomes and 100,000 loci per chromosome. The mutation rate was set to 1 × 10^‐5^ per loci taking as a guide from the mutation rate in maize which was previously estimated to be between 1 × 10^‐4^ and 4.9 × 10^‐6^ (Drake, Charlesworth, Charlesworth, & Crow, 1998). Recombination was sampled from a Poisson distribution with lambda equal to one. The A genome evolution was set at generation 6,000, the B genome at generation 6,200, and the D genome at generation 11,000, and all of them had 500 individuals (population size). The hybridization between the A and B genomes was set at generation 12,000 with a population size equal to 500, while the targeted hexaploid population with 482 individuals evolved at generation 18,000 by hybridizing the tetraploid (AB) population with the D genome. The PolySim simulation was run for 25,000 generations. This number of generations was selected to be sufficient to ensure the progenitor population reached mutation–drift equilibrium before evolving new taxa and at the end of the simulation after 25,000 generations. Each new taxon started with one individual at its first generation and reached the target population size within 100 generations.

After running the previous simulation for 25,000 generations, the hexaploid population was randomly split into two subpopulations, one with 259 individuals representing the Pre70 subpopulation and the other with 223 individuals representing the Post70 subpopulation. Extra generations for each subpopulation were run with restricted gene flow between both subpopulations, while tracking the differentiation between them (*F*_st_) after each generation (Hayes et al., 2009). The simulation stopped when it reached the empirical *F*_st_ between both subpopulations, which was previously calculated as 0.13 (Joukhadar et al., 2017). Ascertainment bias was simulated to mimic the development of the 90K SNP chip (Wang et al., 2014), which was developed using 19 bread wheat cultivars and 18 durum accessions. To do this, 19 individuals were randomly selected from the Post70 subpopulation and 9,858 random polymorphic loci in these 19 individuals were selected to simulate the 90K SNP genotyping (the same number of SNPs used in this study). The genotyping for the whole population (Post70 and Pre70) was then assumed to be conducted using these selected loci only. We also simulated ascertainment bias using 19 individuals from the Post70 population and 18 tetraploid genotypes to exactly mimic the discovery panel used in Wang et al. (2014). However, this analysis did not show any significant difference to the results generated from simulations performed using only the 19 individuals from the Post70 subpopulation (data not shown).

For the analysis of the state‐by‐state comparison, we ran five different analyses corresponding to each of the Australian states used in this study. For each state, individuals were selected from each simulated subpopulation (Pre70 and Post70) to match the number of Pre70 and Post70 individuals within each state. Next, additional generations for each of the five analyses were run to match the *F*_st_ between the state and the remaining states. All selection statistics were then applied on each of the previous six pairs of subpopulations (Pre70 and Post70 plus five states) using the same parameters applied on the empirical subpopulations. Significance thresholds were set at the 99th percentile of the neutral values for XPCLR, iHS, and nSL. Given the high variance of the *F*_st_ values, a more stringent threshold of the 99.9th percentile was applied.

Before running the previous one hundred replicates, we ran multiple simulations to define the best cross‐pollination rate that produced similar LD patterns between the empirical data and our simulated data. Different studies reported outcrossing rates for wheat ranging from 0% to 10.6% (Hanson, Mallory‐Smith, Shafii, Thill, & Zemetra, 2005; Lawrie, Matus‐Cadiz, & Hucl, 2006). We first ran ten simulations using an outcrossing rate between 0.01 and 0.1 and a step equal to 0.01. This analysis revealed that the best LD match was a value between 0.03 and 0.04. We then ran another nine simulations with a step equal to 0.001 between 0.03 and 0.04 to define the optimal outcrossing rate.

### 2.6 Linkage disequilibrium analysis

Pairwise linkage disequilibrium (LD) between the amplicon resequencing alleles and 90K SNPs located on the same chromosome was calculated, as *r* ^2^, following Hill and Robertson (1968) using the R package “*snpstats*” (Clayton, 2015). Similarly, LD was assessed between the amplicon resequencing alleles and candidate regions for artificial selection. Joukhadar et al. (2017) estimated that the 99th percentile of the *r* ^2^ values of the background LD, LD between unlinked SNPs on different chromosomes, is equal to 0.161. Pairwise candidate regions for artificial selection were considered in LD if more than half of the SNPs in each region had an interregion LD higher than the 99th percentile of the background LD. Individual SNPs with very high LD between both regions that did not follow the general LD pattern were excluded to avoid mapping errors. Candidate regions that had less than five SNPs were also excluded from this analysis. The highest *r* ^2^ value from the remaining representative SNPs was reported.

## 3 RESULTS

### 3.1 Simulated Australian wheat germplasm

**Figure 1:**
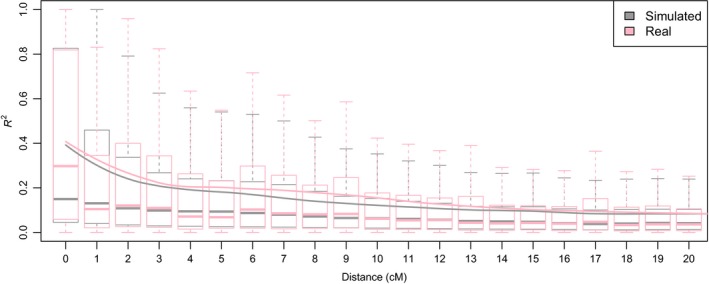
LD decay comparison between simulated and empirical populations

To account for genetic drift and any ascertainment bias in the iSelect 90K SNP genotyping assay, simulations were performed to closely mimic the genomic architecture of the Australian wheat germplasm used in this study. After testing multiple values, an outcrossing rate equal to 0.033 was found to produce the best approximation of linkage disequilibrium decay between the simulated and empirical datasets (Figure 1). In both the simulated and empirical populations, LD started at 0 cM with an *r* ^2^ value of ~0.4 and decayed to an *r* ^2^ of ~0.3 at 1 cM. The heterozygosity (He) in the simulated data was equal to 0.02 ± 0.006, which, as we discuss later, is an acceptable value for our germplasm. The simulation required 35.2 ± 4.7 generations to differentiate the Pre70 from the Post70 cultivars with an *F*_st_ value equal to 0.13 ± 0.002. The number of generations required to differentiate the cultivars released in each state from those released in the other states by the corresponding *F*_st_ value for each state ranged from 5.2 ± 2.3 for New South Wales (NSW) to 15.6 ± 3.6 for Queensland (QLD).

### 3.2 Selection signatures in Australian wheat

**Figure 2:**
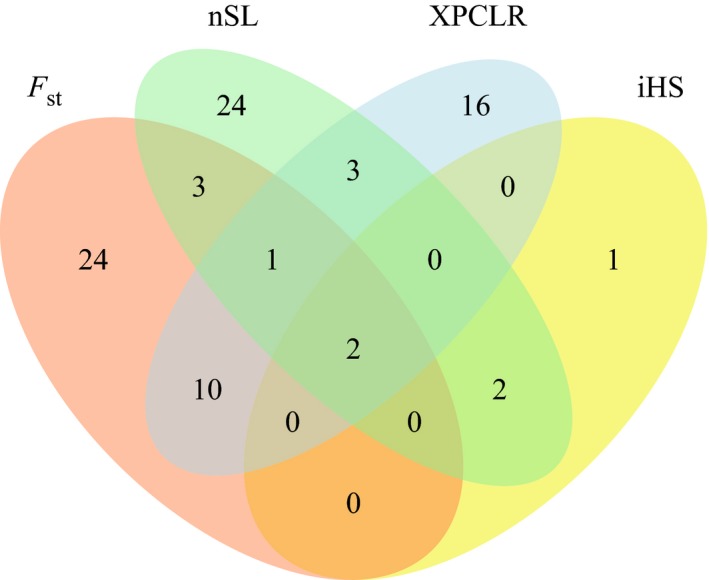
Venn diagram for the selection signature detected using nSL, iHS, XPCLR, and *F*_st_

A total of 86 genomic regions exhibiting putative selection signatures were detected in the Post70 cultivars and in all Australian states using the *F*_st_, nSL, iHS, and XPCLR methods (Table 1; Figure 2). These regions were distributed on all wheat chromosomes except for chromosome 6D, with a maximum of seven regions on chromosomes 1B, 5B, and 6B. The sizes of the selected regions averaged 4.1 cM in length, with a maximum of 34.3 cM for the region on chromosome 3B between genetic map positions 259.1 and 293.4 cM. *F*_st_ identified the largest number of regions with 40 candidates, followed by nSL and XPCLR with 35 and 32, respectively (Figure 2). Twenty‐four selected regions were detected with *F*_st_ only, of which 18 were identified in the Post70 analysis. The LD‐based method iHS identified the smallest number of selection candidates with only five regions detected. Two regions on chromosomes 1B and 7A were detected by all methods, while *F*_st_ and XPCLR had the largest number of candidate regions in common with 13 selective regions (Table 1; Figure 2).

**Table 1:** Genomic regions (chromosome, and start and end positions in centimorgans) under artificial selection detected in different Australian states or time periods using *F*_st_, nSL, iHS, and XPCLR and overlapping genes/QTL within these regions

Region	Chr	PosStart	PosEnd	Pops	Analysis	Gene/QTL	References
1A−216.5:217.2	1A	216.5	217.2	Post70, QLD, SA	*F*_st_, nSL	*QPh, QSe, QKps, QSpad*	Bennett, Reynolds, et al. (2012), Bennett, Izanloo, Reynolds, et al. (2012), Sukumaran, Dreisigacker, et al. (2015), Valluru et al. (2017)
1A−241.9:241.9	1A	241.9	241.9	Post70	nSL	–	–
1A−256.1:256.1	1A	256.1	256.1	WA	XPCLR	*LD with Glu‐A1*	–
1A−289.1:297.4	1A	289.1	297.4	Post70	*F*_st_, XPCLR	–	–
1A−351.3:357.3	1A	351.3	357.3	Post70	*F*_st_	–	–
1B−142:142.9	1B	142.0	142.9	QLD, WA	nSL	–	–
1B−195.4:227.2	1B	195.4	227.2	Post70, All, QLD, SA, VIC, WA	All	*Glu‐B1u, QSe, QAD_Yld*	Sukumaran, Reynolds, et al. (2015), Valluru et al. (2017)
1B−238.3:240.6	1B	238.3	240.6	QLD, VIC	*F*_st_, nSL	–	–
1B−255.1:260.8	1B	255.1	260.8	VIC	*F*_st_, XPCLR	LD with *Glu‐B1c,e*	–
1B−306.5:306.5	1B	306.5	306.5	QLD	*F*_st_	–	–
1B−355.4:368.3	1B	355.4	368.3	Post70, QLD	*F*_st_	–	–
1B−431.2:449.8	1B	431.2	449.8	SA	*F*_st_, XPCLR	–	–
1D−108.9:120.6	1D	108.9	120.6	Post70	*F*_st_	–	–
1D−263.8:264	1D	263.8	264.0	QLD, VIC, WA	iHS, nSL	*QHi?*	Sukumaran, Dreisigacker, et al. (2015)
2A−158:158	2A	158.0	158.0	VIC	XPCLR	–	–
2A−342.7:342.7	2A	342.7	342.7	Post70, QLD	nSL	*QSdw, QW, QPhys*	Bennett, Reynolds, et al. (2012), Valluru et al. (2017)
2A−355.6:363	2A	355.6	363.0	Post70	nSL, XPCLR	*QYld, TaCwi‐A1?*	Bennett, Izanloo, et al. (2012), Mohler et al. (2016), Sukumaran et al. (2018)
2A−413.7:413.7	2A	413.7	413.7	SA	nSL	–	–
2B−84.9:87.4	2B	84.9	87.4	QLD, SA	*F*_st_, XPCLR	*QTkw*	Sukumaran et al. (2018)
2B−216.6:221.6	2B	216.6	221.6	Post70	*F*_st_	*Ppd‐B1?*	–
2B−236.5:236.5	2B	236.5	236.5	NSW	nSL	*–*	–
2B−283.4:291.9	2B	283.4	291.9	VIC	*F*_st_, XPCLR	*QZad, QEet, QW, QEv, QFll*	Bennett, Izanloo, Edwards, et al. (2012); Bennett, Izanloo, Reynolds, et al. (2012); Bennett, Reynolds, et al. (2012)
2B−311.4:320.4	2B	311.4	320.4	Post70, SA WA	*F*_st_, nSL, XPCLR	*Sus−2B, QTpa, QTkw, QGn; QFlw*	Bennett, Izanloo, Reynolds, et al. (2012); Bennett, Reynolds, et al. (2012); Sukumaran et al. (2018)
2B−504.4:504.4	2B	504.4	504.4	QLD	XPCLR	–	–
2D−60.3:66.6	2D	60.3	66.6	SA, VIC	nSL	–	–
3A−107.2:107.2	3A	107.2	107.2	WA	XPCLR	–	–
3A−285.2:285.2	3A	285.2	285.2	QLD	XPCLR	–	–
3A−321.8:321.8	3A	321.8	321.8	Post70	*F*_st_	–	–
3A−336.6:336.6	3A	336.6	336.6	WA	nSL	–	–
3B−56.5:56.6	3B	56.5	56.6	All comparisons	his	*QHi*	Sukumaran, Dreisigacker, et al. (2015)
3B−132:132	3B	132.0	132.0	VIC	XPCLR	–	–
3B−239.1:250.3	3B	239.1	250.3	Post70, QLD	*F*_st_	*QYld, Q.CT‐gf, QKpsm, QTkw, QNdvi*	Bennett, Reynolds, et al. (2012)
3B−259.1:293.4	3B	259.1	293.4	Post70, QLD, SA, VIC, WA	*F*_st_, XPCLR	*QAD_Yld, QAD_Gn, QSe, QDso, QDst, QBmt, QTkw, QNdvi. QYld*	Bennett, Izanloo, Reynolds, et al. (2012); Maphosa et al. (2013); Sukumaran, Dreisigacker, et al. (2015); Valluru et al. (2017)
3B−324:324	3B	324.0	324.0	QLD	XPCLR	–	–
3D−240.6:240.6	3D	240.6	240.6	VIC	*F*_st_	–	–
3D−283.7:286	3D	283.7	286.0	QLD	*F*_st_, XPCLR	–	–
4A−589.7:589.7	4A	589.7	589.7	VIC	XPCLR	–	–
4B−107.3:107.3	4B	107.3	107.3	Post70	*F*_st_	–	–
4B−180.5:182.5	4B	180.5	182.5	QLD, VIC	nSL	–	–
4D−30.5:30.5	4D	30.5	30.5	VIC	XPCLR	–	–
4D−159.5:159.5	4D	159.5	159.5	WA	XPCLR	–	–
5A−77.8:79.2	5A	77.8	79.2	Post70, All, SA, VIC, WA	nSL	*Psy‐A2*	Colasuonno et al. (2017)
5A−216.7:216.8	5A	216.7	216.8	Post70	*F*_st_	–	–
5A−237.4:237.4	5A	237.4	237.4	SA	nSL	–	–
5A−266.6:266.6	5A	266.6	266.6	QLD	nSL	–	–
5A−460.6:466.8	5A	460.6	466.8	Post70	*F*_st_	LD with *Vrn‐A1, QYld*	Lopes et al. (2015)
5A−690.4:699.7	5A	690.4	699.7	Post70	*F*_st_	–	–
5B−168.3:168.3	5B	168.3	168.3	VIC	XPCLR	–	–
5B−222.6:222.7	5B	222.6	222.7	WA	*F*_st_	*Srp5B?*	–
5B−290.3:290.3	5B	290.3	290.3	VIC	XPCLR	–	–
5B−323.8:324.8	5B	323.8	324.8	Post70	*F*_st_	–	–
5B−377:379	5B	377.0	379.0	WA	*F*_st_	*QEet*	Bennett, Izanloo, Reynolds, et al. (2012)
5B−397.5:397.5	5B	397.5	397.5	VIC	nSL	–	–
5B−420.8:420.8	5B	420.8	420.8	QLD	nSL	–	–
5D−180:194.2	5D	180.0	194.2	Post70, SA, WA	nSL, XPCLR	–	–
5D−214.5:223	5D	214.5	223.0	SA, VIC	nSL	–	–
5D−365.2:365.2	5D	365.2	365.2	SA	nSL	–	–
5D−406.4:410.2	5D	406.4	410.2	SA	nSL	–	–
5D−503.5:513.6	5D	503.5	513.6	Post70	*F*_st_	–	–
6A−32.4:32.4	6A	32.4	32.4	Post70	*F*_st_	–	–
6A−103.7:105.9	6A	103.7	105.9	Post70	*F*_st_	–	–
6A−145:145	6A	145.0	145.0	Post70, VIC	*F*_st_, nSL	–	–
6A−188.9:205.5	6A	188.9	205.5	Post70	*F*_st_	*TaGw2−6A, CCD4, QEv, QSPADLLg, QBM, QYld, QTkw, QTwt, QKpsl, QKpsm, QFlw, QTpa, QWsc, QGpc, Q.Phys, QW*	Bennett, Izanloo, Edwards, et al. (2012), Bennett, Reynolds, et al. (2012); Maphosa et al. (2013); Mohler et al. (2016); Colasuonno et al. (2017); Sukumaran, Dreisigacker, et al. (2015), Sukumaran et al. (2018)
6B−164:164	6B	164.0	164.0	SA	nSL	–	–
6B−202:207.6	6B	202.0	207.6	Post70	*F*_st_	–	–
6B−214.3:214.3	6B	214.3	214.3	SA	nSL	–	–
6B−226.8:237.7	6B	226.8	237.7	QLD	nSL	–	–
6B−255.2:272.3	6B	255.2	272.3	Post70	*F*_st_	*TaGw2−6B?*	Mohler et al. (2016)
6B−377.3:377.3	6B	377.3	377.3	Post70	nSL	–	–
6B−388.3:398	6B	388.3	398.0	Post70	*F*_st_, XPCLR	*Cre8*	–
7A−98.3:98.4	7A	98.3	98.4	QLD	*F*_st_, XPCLR	–	–
7A−123.7:123.8	7A	123.7	123.8	SA	*F*_st_	–	–
7A−371.3:399.2	7A	371.3	399.2	All comparisons	All	*TaSus1−7A, TaSAP1−7A, QGn, QEet, QEv, QKpsm, QZad, QYld, QTkw, QGpc, QFcb, QW*	Bennett, Izanloo, Edwards, et al. (2012), Bennett, Izanloo, Reynolds, et al. (2012), Bennett, Reynolds, et al. (2012)), Maphosa et al. (2013), Mohler et al. (2016), Sukumaran et al. (2018)
7A−436:438.4	7A	436.0	438.4	Post70, QLD	nSL, XPCLR	–	–
7A−624.4:624.6	7A	624.4	624.6	VIC	*F*_st_, XPCLR	–	–
7A−670.6:670.6	7A	670.6	670.6	SA	*F*_st_	*Psy‐A1*	Jayatilake et al. (2013)
7B−229.5:229.8	7B	229.5	229.8	Post70, NSW, QLD, SA, VIC, WA	iHS, nSL	*QEet, QZad, QBmt, QWsc*	Bennett, Izanloo, Edwards, et al. (2012), Bennett, Izanloo, Reynolds, et al. (2012), Maphosa et al. (2013)
7B−328.9:328.9	7B	328.9	328.9	VIC	XPCLR	–	–
7B−427.9:427.9	7B	427.9	427.9	VIC	XPCLR	–	–
7B−448.1:448.1	7B	448.1	448.1	QLD	nSL	–	–
7D−3.5:7.7	7D	3.5	7.7	Post70	*F*_st_	–	–
7D−224.5:224.5	7D	224.5	224.5	Post70	XPCLR	–	–
7D−265.7:265.7	7D	265.7	265.7	VIC	nSL	–	–
7D−279.6:282.9	7D	279.6	282.9	Post70, SA	nSL	–	–
7D−298.9:309.4	7D	298.9	309.4	Post70, NSW, QLD, SA, WA	nSL	*QFll*	Bennett, Reynolds, et al. (2012)
7D−414.5:414.5	7D	414.5	414.5	Post70	XPCLR	–	–

Note

Abbreviations: AD, adaptation to density; BM, biomass; Bmt, bake mixing time; CT‐gf, canopy temperature: grain fill; Dso, dough softening; Dst, dough stability; Eet, ear emergence time; Ev, early vigor; Fcb, flour color b; Fll, flag leaf length; Flw, flag leaf weight; Hi, harvesting index; Gn, grain number; Gpc, grain protein content; Kps, kernels per spike; Kpsl, kernels per spikelet; Kpsm, kernels per square meter; NDVI, normalized difference vegetation index; Ph, plant height; Phys, physiological maturity; Q, QTL; Sdw, spike dry weight; Se, spike ethylene production under heat stress; SPAD, SPAD reading for chlorophyll content; SPADLLg, SPAD at the grain filling stage; Tkw, thousand kernel weight; Tpa, tillers per square meter; Twt, test weight; W, flag leaf glaucousness; Wsc, water‐soluble carbohydrates; Yld, yield; Zad, Zadoks growth score.

**Figure 3:**
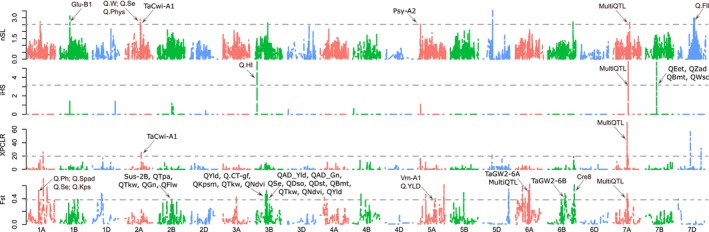
Selection signatures detected in the Post70 population using nSL, iHS, XPCLR, and *F*_st_. Horizontal lines represent the significance thresholds

The largest number of selection signatures was detected for the Post70 cultivars with 39 regions, followed by cultivars based on state of release with QLD, VIC, SA, WA, and NSW exhibiting 26, 26, 22, 17, and 5 regions, respectively (Table 1). Twenty‐three candidate regions for artificial selection were detected in multiple populations. Running the nSL and iHS analyses on the entire population of 482 cultivars resulted in only four selective sweeps, of which one was reported in all analyses except NSW and another for all analyses except NSW and QLD (Table 1). Of the remaining two, the sweep between 371.3 and 399.2 cM on chromosome 7A and that between 195.4 and 227.2 cM on chromosome 1B were reported in all the analyses performed. Interestingly, differentiation‐based methods (*F*_st_ and XPCLR) did not detect any candidate regions for selection in NSW. Figure 3 shows a Manhattan plot for the four methods applied on the Post70 population, while the remaining comparisons can be found in Figure S1.

### 3.3 Amplicon resequencing

Several characterized alleles showed different patterns of allele frequencies between the pre‐ and post‐Green Revolution cultivars, or when compared across different Australian states indicating differential selection on them (Table 2). The frequency of the resistance allele at the *Cre8* locus which confers resistance to CNN was significantly increased (85.5% frequency) in the Post70 population and had a significant *F*_st_ value (0.4), compared to the Pre70 population. The frequency of the Green Revolution alleles *Rht‐B1b* and *Rht‐D1b*, which affect plant height, was also significantly increased (0.55 and 0.39) in the Post70 cultivars and had *F*_st_ values of 0.89 and 0.6, respectively. Their frequency increased to 0.94 when Post70 cultivars having only one *Rht* allele (*Rht‐B1b* or *Rht‐D1b*) were considered. Interestingly, the *Rht‐D1b* allele appeared to be more favored in SA (Table 2). The frequency of the allele *Ppd‐D1a* genes was significantly increased (*F*_st_ = 1) over the *Ppd‐D1d* allele in the Post70 cultivars, which significantly decreased (*F*_st_ = 0.67). However, this was not the case for SA in which the frequency of the *Ppd‐D1d* allele significantly increased with *F*_st_ = 0.16. The *F*_st_ of the high‐activity allele of SUCROSE SYNTHASE (*Sus‐2B_H*), which reversibly catalyzes sucrose synthesis and cleavage and represents a key enzyme in the control of the flow of carbon into starch biosynthesis, showed a low‐to‐moderate but not significant increase in all comparisons except for SA where it was significantly decreased with a *F*_st_ value of 0.22. The frequencies for the three homoeologous vernalization genes (*Vrn‐A1, Vrn‐B1*, and *Vrn‐D1*) varied among states, while the Post70 cultivars showed higher but not significant *F*_st_ for the *Vrn‐A1 *and *Vrn‐D1 *homoeologs of the vernalization insensitive (*a*) alleles. The allele *Vrn‐A1b* had an increased frequency in VIC. For QLD, *Vrn‐D1a* had an increased *F*_st_ (but not significant), while the *F*_st_ of the *Vrn‐B1a* allele was significantly decreased.

**Table 2:** Allele frequency and *F*_st_ (between brackets) results for major alleles of the 18 characterized genes. Yellow‐highlighted values represent alleles that got significantly increased, while orange‐highlighted values represent alleles that got significantly decreased. Best LD describes the position (in centimorgans) on the same chromosome that has the highest LD with the characterized allele

Gene	Allele	Chr	Best LD	*r* ^2^	Freq	Post−1970	NSW	QLD	SA	VIC	WA
*Cre8* ^a^	R	6B	398.0	0.40	60.5	85.5 (0.4)	70.2 (0.04)	69 (0.04)	50 (0.04)	49.4 (0.05)	60.6 (0)
*Glu‐A1*	A	1A	260.4	0.50	56.9	51.6 (0.02)	56.2 (0)	56.3 (0)	70.3 (0.08)	67.5 (0.05)	32.1 (0.24)
*Glu‐A1*	B	1A	264.6	0.59	35.7	39.3 (0.01)	34.6 (0)	32.4 (0.01)	27 (0.04)	20.8 (0.1)	66.7 (0.4)
*Glu‐A1*	C	1A	260.4	0.64	7.4	9.1 (0.01)	9.2 (0.01)	11.3 (0.02)	2.7 (0.03)	11.7 (0.03)	1.2 (0.05)
*Glu‐B1*	C	1B	260.9	0.38	26.6	15.8 (0.07)	22.9 (0.01)	32.3 (0.02)	39.6 (0.09)	11.4 (0.11)	25.3 (0)
*Glu‐B1*	E	1B	260.9	0.91	28.2	4.6 (0.41)	22.9 (0.01)	4.6 (0.28)	36.5 (0.03)	57.1 (0.41)	20.3 (0.03)
*Glu‐B1*	F	1B	251.0	0.51	3.3	6.1 (0.05)	2.5 (0)	1.5 (0.01)	0 (0.03)	1.4 (0.01)	11.4 (0.2)
*Glu‐B1*	I	1B	251.0	0.73	24.2	37.2 (0.16)	32.2 (0.04)	29.2 (0.02)	12.5 (0.07)	11.4 (0.09)	34.2 (0.06)
*Glu‐B1*	U	1B	230.3	0.60	17.7	36.2 (0.43)	19.5 (0)	32.3 (0.14)	11.5 (0.03)	18.6 (0)	8.9 (0.06)
*Glu‐B3*	B	1B	35.5	0.52	61.1	63.4 (0)	65.5 (0.01)	73.1 (0.06)	59.8 (0)	49.3 (0.06)	56.1 (0.01)
*Glu‐B3*	C	1B	31.3	0.58	9.2	1.4 (0.09)	10.9 (0)	0 (0.1)	5.9 (0.01)	24.7 (0.28)	4.9 (0.02)
*Glu‐B3*	F	1B	NA	NA	2.7	0.5 (0.03)	1.7 (0)	4.5 (0.01)	2.9 (0)	2.7 (0)	2.4 (0)
*Glu‐B3*	G	1B	35.5	0.75	7.0	3.8 (0.03)	5.9 (0)	1.5 (0.05)	7.8 (0)	11 (0.02)	8.5 (0)
*Glu‐B3*	H	1B	52.7	0.25	16.4	26.8 (0.16)	15.1 (0)	17.9 (0)	19.6 (0.01)	9.6 (0.03)	19.5 (0.01)
*Glu‐B3*	I	1B	NA	NA	3.5	4.2 (0)	0.8 (0.02)	2.9 (0)	3.8 (0)	2.7 (0)	8.3 (0.06)
*Glu‐D1* ^a^	A	1D	145.2	0.86	61.9	68.2 (0.02)	61.1 (0)	67.6 (0.01)	49.5 (0.08)	63.2 (0)	72.9 (0.04)
*PinA* ^a^	A	5D	270.6	0.41	81.6	73.7 (0.09)	77.5 (0.01)	75 (0.03)	86.2 (0.01)	90.8 (0.05)	79.8 (0)
*PinB* ^a^	A	5D	264.3	0.81	63.2	47.3 (0.19)	56.9 (0.02)	49.3 (0.08)	65.8 (0)	73.3 (0.04)	70.6 (0.02)
*PinA/PinB*	One copy	5D	–	–	55.4	78.5 (0.19)	65.6 (0.05)	75 (0.17)	48.1 (0.02)	36.8 (0.13)	47.5 (0.02)
*Ppo‐A1* ^a^	A	2A	411.8	0.79	62.6	80.4 (0.21)	69.4 (0.03)	62.9 (0)	70.2 (0.03)	48.6 (0.07)	55.8 (0.01)
*Ppo‐D1* ^a^	A	2D	215.9	0.81	83.1	74.6 (0.11)	86.9 (0)	92.3 (0.05)	70.8 (0.15)	92 (0.04)	81.2 (0.01)
*Psy‐A1*	A	7A	663.9	0.46	41.0	33.9 (0.04)	47.3 (0.02)	29.6 (0.05)	36.4 (0.01)	47.3 (0.02)	41.8 (0)
*Psy‐A1*	B/E	7A	663.9	0.31	15.7	32.8 (0.42)	19 (0)	31 (0.15)	5.5 (0.09)	17.6 (0)	10.6 (0.03)
*Psy‐A1*	C	7A	NA	NA	3.4	2.5 (0)	5 (0.01)	1.4 (0.01)	0.9 (0.02)	0 (0.04)	10 (0.13)
*Psy‐A1*	P/Q	7A	670.6	0.47	29.3	16.3 (0.11)	23.3 (0.03)	35.9 (0.01)	30 (0)	33.8 (0)	30.6 (0)
*Psy‐A1*	R	7A	663.9	0.38	3.6	5 (0.01)	0.8 (0.02)	0 (0.03)	10.9 (0.21)	0 (0.03)	3.5 (0)
*Psy‐A1*	S/T	7A	670.6	0.84	6.2	9.4 (0.02)	4.7 (0)	2.1 (0.02)	16.4 (0.22)	1.4 (0.03)	3.5 (0.01)
*Wx‐B1* ^a^	A	4A	430.7	0.34	58.1	53.5 (0.02)	54.7 (0.01)	57.7 (0)	52.8 (0.01)	71.1 (0.06)	57 (0)
*Ppd‐D1*	A	2D	NA	NA	29.6	63.2 (1)	25.2 (0.02)	47.9 (0.13)	16.2 (0.1)	28 (0)	38.8 (0.03)
*Ppd‐D1*	B	2D	NA	NA	17.5	16.1 (0)	28.6 (0.1)	19.7 (0.01)	12.2 (0.02)	7.3 (0.06)	16.5 (0)
*Ppd‐D1*	D	2D	NA	NA	52.5	20.7 (0.67)	46.2 (0.01)	32.4 (0.15)	71.6 (0.16)	64.7 (0.07)	44.7 (0.02)
*Rht‐B1* ^a^	B	4B	162.1	0.63	27.1	54.7 (0.89)	25.8 (0)	37.1 (0.03)	13 (0.12)	35.8 (0.02)	32.9 (0.01)
*Rht‐D1* ^a^	B	4D	525.3	0.29	17.8	39.2 (0.6)	14.4 (0)	14.4 (0)	27.5 (0.08)	6 (0.09)	23 (0.02)
*Rht‐B1/Rht‐D1*	One copy	4B/4D	–	–	46.3	94.0 (0.89)	43.8 (0)	52.5 (0.01)	40.8 (0.01)	40.6 (0.02)	56.2 (0.04)
*Sus−2B* ^a^	H	2B	319.8	0.36	59.5	74.2 (0.14)	61.7 (0)	67.6 (0.02)	38.2 (0.22)	66.2 (0.01)	70.6 (0.04)
*Vrn‐A1*	A	5A	457.1	0.65	30.8	47.4 (0.23)	25.6 (0.02)	32.9 (0)	34 (0)	34.2 (0)	31.6 (0)
*Vrn‐A1*	B	5A	421.2	0.38	32.0	9.1 (0.36)	29.6 (0)	12.9 (0.16)	44.3 (0.08)	50.7 (0.17)	20.3 (0.06)
*Vrn‐A1*	V	5A	450.4	0.36	37.0	43.5 (0.03)	44.8 (0.03)	54.3 (0.13)	21.7 (0.1)	15.1 (0.2)	48.1 (0.06)
*Vrn‐B1*	A	5B	340.3	0.43	67.5	52.1 (0.18)	57.8 (0.04)	38.7 (0.37)	83.9 (0.13)	74.7 (0.03)	81 (0.09)
*Vrn‐B1*	B	5B	NA	NA	3.2	6.2 (0.05)	2.3 (0)	2.8 (0)	5 (0.01)	2.7 (0)	3.6 (0)
*Vrn‐B1*	V	5B	340.3	0.43	29.2	41.7 (0.13)	39.9 (0.05)	58.5 (0.4)	11 (0.16)	22.7 (0.02)	15.5 (0.09)
*Vrn‐D1* ^a^	A	5D	NA	NA	11.7	18.5 (0.07)	13.9 (0)	27.5 (0.2)	6.5 (0.04)	8.2 (0.02)	7.6 (0.02)

^a^ Genes that have only two alleles

The frequencies of alleles for the ten quality‐related genes were also variable in the different subpopulations. For *Psy‐A1*, alleles *Psy‐A1b,e* showed a significantly increased *F*_st_ in the Post70 subpopulation. The *F*_st_ of allele *Psy‐A1c *was significantly higher in WA, while alleles R and *Psy‐A1s,t *were higher in SA. The *Ppo‐D1a* allele was significantly decreased *F*_st_ in SA, while *Ppo‐A1* and *Wx‐B1* showed no significant difference in any subpopulation. The two adjacent grain hardness genes on chromosome 5D (*PinA* and *PinB*) also showed no significant difference for any comparison, but the combination of both alleles—representing one hard allele—showed a significant decrease in VIC. The haplotype that carried the two hard alleles did not exist in any of the studied Australian cultivars, indicating a strong selection against this haplotype. For the glutenin genes, the low molecular weight glutenin subunit gene (*Glu‐B3*) showed a significant frequency increase for the allele *Glu‐B3c* in VIC, while no significant increase was detected for the remaining alleles, although allele *Glu‐B3h* showed a considerable increase in the Post70 cultivars. For the high molecular weight glutenin subunit genes, the frequency of the allele *Glu‐A1b* increased significantly in WA with *F*_st_ = 0.4, while none of the *Glu‐D1* alleles exhibited any significant difference. For *Glu‐B1*, allele *Glu‐B1u* was significantly favored over allele *Glu‐B1e* in the Post70 subpopulation, while VIC significantly preferred the allele *Glu‐B1e* over the allele *Glu‐B1c*. Allele *Glu‐B1f* significantly increased in WA, and allele *Glu‐B1e* significantly decreased in QLD (Table 2).

### 3.4 Linkage disequilibrium between characterized alleles and candidate regions under artificial selection

Several characterized alleles with significant differentiation between the pre‐ and post‐Green Revolution cultivars or between states were correlated with genomic regions affected by artificial selection. *Glu‐A1* had the highest LD with SNPs on chromosome 1A at 260.4 cM, but it also had a high LD (*r* ^2^ *^^*= 0.26) with the selective sweep 1A‐256.1:256.1 which was detected only in WA. The position of the *Glu‐B1* gene was within the sweep 1B‐195.4:227.2. However, alleles *Glu‐B1c* and *Glu‐B1e* had high LD (*r* ^2^ = 0.38 and 0.91, respectively) with the adjacent sweep 1B‐255.1:260.8. The sucrose synthase gene (Sus‐2B) had *r* ^2^ = 0.36 with the sweep 2B‐311.4:320.4, while the *Cre8* gene was located within the sweep 6B‐388.3:398 with *r* ^2^ = 0.4. Multiple alleles of the *Psy‐A1* gene had the highest LD with SNPs within the sweep 7A‐670.6:670.6 with the highest *r* ^2^ equal to 0.84. The sweep 5A‐460.6:466.8 had LD with *Vrn‐A1 *which had the highest LD only 3.5 cM away from the sweep at 257.1 cM.

Another interesting finding was 17 unlinked selective sweeps and characterized genes (*Glu‐B1u, Cre8_R, Psy‐A1b&e, Ppd‐D1a, Rht‐B1b*, and *Vrn‐B1a*) with moderate‐to‐high differentiation across different subpopulations that exhibited moderate‐to‐high LD with each other. Many of the sweeps could be potentially affecting grain yield and/or its components as we will discuss later. The average *r* ^2^ among these genomic regions was 0.35, which is equivalent to the 99.97th percentile of the background LD for unlinked SNPs. This result suggests simultaneous and strong selection for specific alleles across the wheat genome (Table 3). Of the 17 sweeps, the regions separating the sweeps located on chromosomes 3B and 6A had no or weak LD with the regions under artificial selection.

**Table 3:** *r* ^2^ values for between the 17 unlinked genomic regions and characterized alleles that showed moderate‐to‐high correlation with each other

Region/Allele	1A−216.5:217.2	1B−195.4:227.2 (*Glu‐B1*)	1B−355.4:368.3	3B−056.5:056.6	3B−239.1:250.3	3B−259.1:293.4	5A−216.7:216.8	5D−503.5:513.6	6A−032.4:032.4	6A−103.7:105.9	6A−188.9:205.5 (*TaGw2−6A*)	6B−388.3:398.0 (*Cre8*)	7A−371.3:399.2 (*TaSus1−7A, TaSAP1−7A*)	*Ppd‐D1*	*Rht‐B1*	*Vrn‐B1*	*Psy‐A1*
1A−216.5:217.2	1.00	0.34	0.22	0.20	0.25	0.38	0.27	0.26	0.28	0.35	0.36	0.45	0.39	0.39	0.32	0.17	0.20
1B−195.4:227.2 (*Glu‐B1*)	0.34	1.00	0.51	0.32	0.46	0.46	0.43	0.33	0.28	0.28	0.41	0.31	0.43	0.23	0.25	0.23	0.44
1B−355.4:368.3	0.22	0.51	1.00	0.42	0.49	0.49	0.42	0.28	0.22	0.35	0.53	0.36	0.54	0.31	0.39	0.23	0.38
3B−056.5:056.6	0.20	0.32	0.42	1.00	0.34	0.37	0.42	0.23		0.22	0.42	0.25	0.40	0.15	0.31	0.15	0.32
3B−239.1:250.3	0.25	0.50	0.49	0.34	1.00	0.75	0.40	0.31	0.37	0.37	0.52	0.29	0.53	0.26	0.39	0.22	0.32
3B−259.1:293.4	0.38	0.53	0.49	0.37	0.75	1.00	0.40	0.44	0.30	0.56	0.47	0.52	0.53	0.46	0.44	0.25	0.30
5A−216.7:216.8	0.27	0.43	0.42	0.42	0.40	0.40	1.00	0.22	0.25	0.39	0.50	0.37	0.47	0.35	0.57	0.17	0.36
5D−503.5:513.6	0.26	0.33	0.28	0.23	0.31	0.44	0.22	1.00	0.44	0.39	0.30	0.36	0.42	0.30	0.21	–	0.16
6A−032.4:032.4	0.28	0.28	0.22	–	0.37	0.30	0.25	0.44	1.00	0.44	0.26	0.32	0.34	0.26	0.21	–	0.18
6A−103.7:105.9	0.35	0.28	0.35	0.22	0.37	0.56	0.39	0.39	0.44	1.00	0.40	0.55	0.50	0.50	0.40	0.17	0.25
6A−188.9:205.5 (*TaGw2−6A*)	0.36	0.41	0.53	0.42	0.52	0.47	0.50	0.30	0.26	0.40	1.00	0.43	0.40	0.25	0.37	0.19	0.22
6B−388.3:398.0 (*Cre8*)	0.45	0.31	0.36	0.25	0.29	0.52	0.37	0.36	0.32	0.55	0.43	1.00	0.58	0.54	0.39	–	0.25
7A−371.3:399.2 (*TaSus1−7A, TaSAP1−7A*)	0.39	0.47	0.54	0.40	0.53	0.53	0.47	0.42	0.34	0.50	0.40	0.58	1.00	0.45	0.41	0.21	0.26
*Ppd‐D1*	0.39	0.27	0.31	0.15	0.26	0.46	0.35	0.30	0.26	0.50	0.25	0.54	0.45	1.00	0.44	0.15	0.24
*Rht‐B1*	0.32	0.25	0.39	0.31	0.39	0.44	0.57	0.21	0.21	0.40	0.37	0.39	0.41	0.44	1.00	0.20	0.44
*Vrn‐B1*	0.17	0.23	0.23	0.15	0.22	0.25	0.17	–	–	0.17	0.19	–	0.21	0.15	0.20	1.00	0.18
*Psy‐A1*	0.20	0.44	0.38	0.32	0.32	0.30	0.36	0.16	0.18	0.25	0.22	0.25	0.26	0.24	0.44	0.18	1.00

## 4 DISCUSSION

### 4.1 Simulations to determine significance thresholds for selection analyses

To address the known problems when choosing appropriate significance thresholds for genomewide selection scans (Kelley et al., 2006; Teshima et al., 2006), we simulated the evolution of the Australian wheat population assuming neutrality to differentiate the effect of random genetic drift from true selection signatures to determine significance thresholds specific to the population used in this study. The simulations were performed using the PolySim software, which takes into account the organism's polyploidy and self‐pollination. Simulation can offer a more precise detection for the significance threshold than the commonly used outlier approaches (Pavlidis & Alachiotis, 2017). For example, in NSW cultivars, no selection signatures were detected using differentiation‐based methods in this study, which is expected given the variety of climates in the NSW wheat‐growing zone (climates in north NSW are similar to QLD, while south NSW is similar to VIC and SA), and the frequent use of wheat cultivars developed in NSW in the breeding programs of other Australian states. Joukhadar et al. (2017) showed that NSW had the lowest *F*_st_, with the remaining states (*F*_st_ between 0.01 and 0.03), while the *F*_st_ values between the remaining states were between 0.03 and 0.08. Thus, outlier approaches would result with 100% false‐positive rate in such populations.

To assess the accuracy of the simulation, we looked at how well the simulated genomic architecture (in terms of LD and He) matched that observed in the population of 482 Australian cultivars. Matching the genomic architecture (usually LD and He) between the simulated and empirical datasets is reported to be the best determinant for measuring the precision of simulated populations used for different purposes (Jighly et al., 2018). The differences between the LD decay smoothing lines of the empirical and simulated populations were minor (Figure 1) and fell within the standard deviation limits of the simulated populations, indicating high similarity for LD decay between the simulated and empirical datasets. A direct assessment of He between the simulated and empirical datasets could not be made due to limitations in calling heterozygous genotypes for hexaploid wheat using the iSelect 90K SNP genotyping assay (Wang et al., 2014). This is because of the large number of clusters produced by the genotype calling software (given the duplicated targets for the hybridization probes in polyploid species) and the low density of the clusters representing heterozygote genotypes in self‐pollinated crops. However, it is possible to infer the accuracy for He, given that wheat breeders usually release cultivars after several generations (six or more) of self‐pollination to ensure a stable cultivar with high homozygosity. This leads to an expected empirical He equal or less than 3.125% (i.e., 1/2^6−1^) for a polymorphic locus, where 6 is the number of selfed generations. Therefore, the simulated He should be acceptable given that its 95% confidence interval was between 3.2% and 0.8%, which is almost equivalent to six to eight generations of selfing. When simulating ascertainment bias, including durum genotypes in the ascertained panel did not have any significant effect on the significance threshold possibly because durum is a common ancestor for both Pre70 and Post70 populations.

### 4.2 Genomic regions under artificial selection

Candidate genomic regions for artificial selection were compared with known genes and published QTL in different Australian wheat or CIMMYT germplasm. These populations should be genetically related to the Australian wheat population used in this study to avoid false‐positive overlapping between the genomic regions under artificial selection and previously reported QTL, considering the huge number of mapping studies on hexaploid wheat (McIntosh et al., 2013). Sixty‐one regions had no overlap with previously reported genes/QTL and thus represent potential novel regions under selection, and it will be of interest to further understand their contribution to the evolution and adaptation of the Australian wheat population.

Cultivars that are less affected by nearby plant competition were reported to have higher yield potential (Reynolds, Acevedo, Sayre, & Fischer, 1994). It was previously proposed that a large proportion of CIMMYT post‐Green Revolution cultivars’ potential is due to their adaptability to planting density (Reynolds et al., 1994). The two QTL with the highest significance and *r* ^2^ from the three QTL reported in Sukumaran, Reynolds, Lopes, and Crossa (2015) that were associated with adaptation to agronomic planting density on chromosomes 1B and 3B exhibited very strong selective sweeps in the germplasm used in this study. Both sweeps were found in multiple populations, and the 1B sweep was detected with both differentiation and haplotype‐based methods indicating their robustness. The 1B sweep region also included *Glu‐B1 *and a QTL for spike ethylene production under heat stress, while the 3B had multiple yield and yield component‐related QTL, dough softening and stability, bake mixing time, spike ethylene, and adaptation to density for grain number QTL (Bennett, Izanloo, Reynolds, et al., 2012; Maphosa et al., 2013; Sukumaran, Dreisigacker, Lopes, Chavez, & Reynolds, 2015; Sukumaran, Reynolds, et al., 2015; Valluru, Reynolds, Davies, & Sukumaran, 2017). Ethylene production can be induced under heat stress, which limits grain yield (Hays, Do, Mason, Morgan, & Finlayson, 2007). Given the large number of potential genes targeted by artificial selection, these sweeps were the largest two sweeps in this study spanning 34.3 cM and 31.8 cM for the 3B and 1B sweeps, respectively. The third largest sweep identified here was the region 7A‐371.3:399.2 spanning 27.9 cM and was detected in all subpopulations using all statistical tests. This region encompasses the genes *TaSus1‐7A* and *TaSAP1‐7A* as well as a number of yield, different yield components, and quality QTL (Bennett, Izanloo, Edwards, et al., 2012; Bennett, Reynolds, et al., 2012; Maphosa et al., 2013; Mohler et al., 2016; Sukumaran, Lopes, Dreisigacker, & Reynolds, 2018). The average size of LD blocks in this germplasm was previously reported to be ~19 cM (Joukhadar et al., 2017). Thus, it is expected that selection may cause such large LD blocks in our germplasm.

In addition to the selective sweeps described earlier, six genomic regions under selection on chromosomes 2A, 2B, 3B, 5A, 6A, and 6B were colocated with genes or QTL that directly affect grain yield and some other traits. These six regions were detected in the Post70 population as well as in some states (Table 1). The 6A region involved the well‐documented grain yield gene *TaGw2‐6A*, while the 6B region could potentially be the homoeologous gene *TaGw2‐6B* (Mohler et al., 2016). The 6A sweep was previously reported in multiple studies to be associated with different yield and yield component traits as well as some quality traits such as grain protein content and water‐soluble carbohydrates (Bennett, Reynolds, et al., 2012; Maphosa et al., 2013; Sukumaran, Dreisigacker, et al., 2015; Sukumaran et al., 2018). The 9‐cis‐epoxycarotenoid dioxygenase (*CCD4* or *NCED4*) gene, which plays an important role during heat stress and thermoinhibition of seeds (Huo, Dahal, Kunusoth, McCallum, & Bradford, 2013), was also in this region (Colasuonno et al., 2017). The region 5A‐460.6:466.8 had LD with *Vrn‐A1*, and Lopes, Dreisigacker, Peña, Sukumaran, and Reynolds (2015) reported a grain yield QTL in this region. The yield QTL located within the sweep 2A‐355.6:363 is most probably related to the cell wall invertase gene *TaCwi‐A1* (Bennett, Izanloo, Reynolds, et al. 2012; Mohler et al., 2016; Sukumaran et al., 2018). The selective sweep 2B‐311.4:320.4 involved the sucrose synthase 2 gene *Sus‐2B* which is colocated with QTL for tillers per square meter, thousand kernel weights, grain number, and flag leaf width (Bennett, Izanloo, Reynolds, et al. (2012); Bennett, Reynolds, et al. (2012); Sukumaran et al., 2018). The characterization results of the *Sus‐2B* gene also showed significant differentiation for its causal variant in SA similar to this sweep (Table 2).

There were also nine other regions on chromosomes 1A, 1D, 2A, 2B, 3B, 5B, 7B, and 7D that affect grain yield indirectly through affecting one or more yield components (Table 1). For example, the selective sweeps 1D‐263.8:264 and 3B‐56.5:56.6 were regions affecting harvesting index, while the sweep 2B‐84.9:87.4 involved a QTL for thousand kernel weights (Sukumaran, Dreisigacker, et al., 2015; Sukumaran et al., 2018). The sweep 7B‐229.5:229.8 affected two yield components, ear emergence time and Zadoks growth score, as well as two quality traits, bake mixing time and water‐soluble carbohydrates (Bennett, Izanloo, Edwards, et al., 2012; Maphosa et al., 2013), while the sweeps 1A‐216.5:217.2 had a QTL for spike ethylene production under heat stress (Valluru et al., 2017). Interestingly, all major genes and yield‐related QTL were detected with *F*_st_/XPCLR indicating hard selection on yield genes and QTL.

As discussed before, many of the previous grain yield or yield‐related sweeps involved quality‐related traits such as the sweep 1B‐195.4:227.2, which involved the glutenin gene *Glu‐B1*. The flour color genes *Psy‐A1* (Jayatilake et al., 2015) and *Psy‐A2* (Colasuonno et al., 2017) that are important for flour quality were also under artificial selection in our germplasm. Previous reports on Australian wheat (Crawford et al., 2011) showed that *Psy‐A1a* and *Psy‐A1p* may not be responsible for the variation in flour color and the frequency of both alleles did not have any significant change in any comparison in the present study. The *Psy‐A1e* allele was previously reported to be common in Australia and responsible for white flour color, while the *Psy‐A1c, Psy‐A1r*, and *Psy‐A1s,t* were associated with cream‐to‐yellow color (Howitt et al. 2009; Crawford et al., 2011). Although the white flour color allele *Psy‐A1e* has become significantly more common after 1970, alleles responsible for cream‐to‐yellow color had significantly higher frequency in SA and WA. The white and bright flour is preferable for most end products, but the creamy colored flour, which is produced mainly in WA, is preferable for some products like the Japanese Udon noodles. The grain hardness genes (*PinA* and *PinB*) did not significantly differ after the Green Revolution, but the frequency of the presence of a single hardness allele showed a significant decrease in VIC. The *Glu‐B1e* allele that produces weak and extensible dough (Eagles et al., 2006) as well as *Glu‐B3c* that produces weak dough (Eagles, Hollamby, Gororo, & Eastwood, 2002) also showed significant increased frequency in VIC. Victoria is a major producer for the Australian soft wheat which is used for cakes, pastries, and some types of biscuits that require weak and extensible dough (Eagles et al., 2006; Simmonds, 1989).

Other characterized vernalization, photoperiod, and plant height genes (*Vrn‐B1, Rht‐B1, Rht‐D1*, and *Ppd‐D1*) that showed significant differentiation (*F*_st_) in different comparisons in Australian wheat (Table 2) were not detected in any of the selection analyses possibly due to the low SNP coverage around them that can detect all alleles. The combination of different alleles of these genes can be used to optimize the flowering date for different environments. Similarly, Eagles et al. (2009) reported that *Ppd‐D1a, Rht‐B1b*, and *Rht‐D1b* did not exist in Australia before the Green Revolution, while the *Vrn‐A1b* was replaced with *Vrn‐A1a* after the Green Revolution (Table 2). Under southern Australian environmental conditions, the allele *Ppd‐D1d* caused more delayed heading compared to *Ppd‐D1a*. This delay was larger when combined with the spring alleles of the three *Vrn1* homoeologs than when it was combined with *Vrn‐B1a* only indicating epistatic interactions between these genes (Cane et al., 2013). This may explain the significant increase in both *Ppd‐D1d* and *Vrn‐B1a* in SA.

An interesting finding was the moderate‐to‐high linkage disequilibrium among 17 unlinked genomic regions and characterized genes that were the subjects of artificial selection (Table 3). All but one of these regions were detected by the differentiation‐based methods indicating hard selective sweeps. All were detected in the Post70 subpopulation, and some overlapped with the different Australian states (Table 1). These regions involved the two sweeps associated with yield adaptation to agronomic planting density, and one which involved *Glu‐B1*, a known major driver for bread making quality. Another three regions encompassed QTL affecting grain yield, and two regions were associated with QTL affecting different yield components. Of the characterized genes, *Cre8, Psy‐A1, Ppd‐D1, Rht‐B1*, and *Vrn‐B1* were involved in this LD cluster. Several allele combinations for different unlinked height, vernalization, and photoperiod genes have previously been reported to be prevalent in Australian wheat germplasm (Cane et al., 2013; Eagles et al., 2009). Similarly, we found that some of these unlinked genes exhibited moderate‐to‐high LD with one other, as well as with other genes controlling grain quality, disease resistance, and yield potential, indicating the ongoing and simultaneous selection on specific alleles.

## 5 CONCLUSION

The results presented here provide a greater understanding of the selection events that shaped current Australian wheat germplasm. Defining the genes targeted by artificial selection has the potential to further guide the grain industry to adapt new genetic resources with novel genetic variation that are differentiated from the present Australian gene pool to sustain long‐term genetic gain. The simultaneous selection on multiple unlinked genes revealed here has limited the diversity of these genes and their flanking genomic regions. Strategies to reduce this correlation (linkage disequilibrium) should be applied to maintain higher levels of genetic diversity and avoid the severe reduction in the germplasm effective population size. These could include adapting new genetic resources to reduce the dependency on the same genes; improving the diversity of the genomic region by recombining multiple diverse haplotypes flanking the desired genes targeted by selection; and exploiting the natural variation of these genomic regions after editing the gene targeted by selection to produce a desired allele.

## CONFLICT OF INTEREST

The authors declared no competing interests.

## Data Availability

Data for this study are available at the Dryad Digital Repository: https://doi.org/10.5061/dryad.06c67 (Joukhadar, Daetwyler, Bansal, Gendall, & Hayden, 2017).
