# Supplementary material for: Artificial selection causes significant linkage disequilibrium among multiple unlinked genes in Australian wheat
Source: Evol Appl. 2019 Jul 18;12(8):1610–25. doi: 10.1111/eva.12807 (PMC6708422; doi:10.1111/eva.12807)
Supplement: Supplementary file 1 [file EVA-12-1610-s001.pdf]

Figure S1. Selection signatures detected in each of the five Australian states using nSL, iHS, XPCLR and Fst. Horizontal lines represent the significant thresholds.

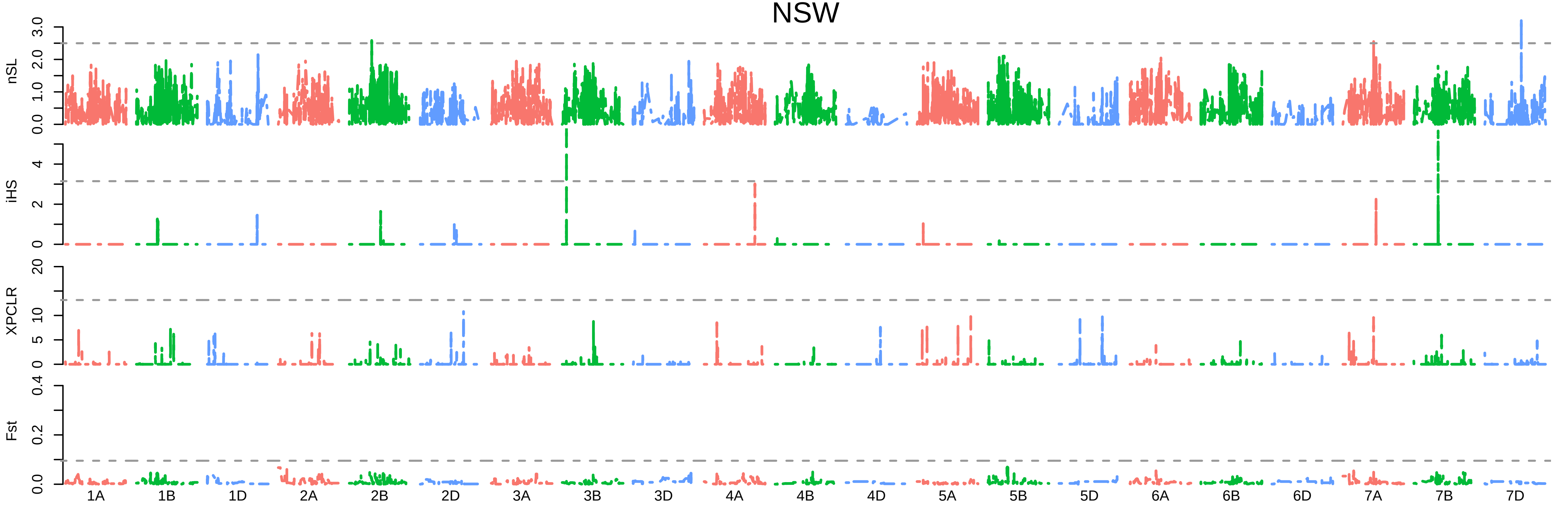

NSW
nSL
0.0
1.0
2.0
3.0
iHS
0
2
4
XPCLR
0
5
10
20
Fst
0.0
0.2
0.4
1A
1B
1D
2A
2B
2D
3A
3B
3D
4A
4B
4D
5A
5B
5D
6A
6B
6D
7A
7B
7D

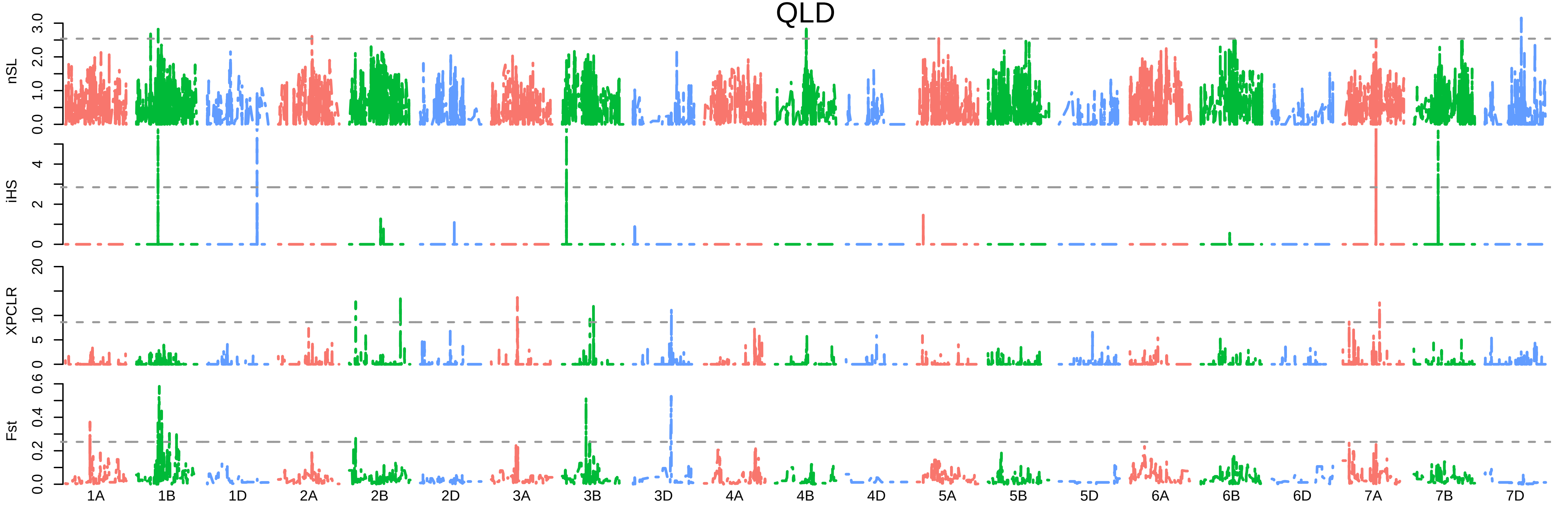
QLD
nSL
0.0
1.0
2.0
3.0
iHS
0
2
4
XPCLR
0
5
10
20
Fst
0.0
0.2
0.4
0.6
1A
1B
1D
2A
2B
2D
3A
3B
3D
4A
4B
4D
5A
5B
5D
6A
6B
6D
7A
7B
7D

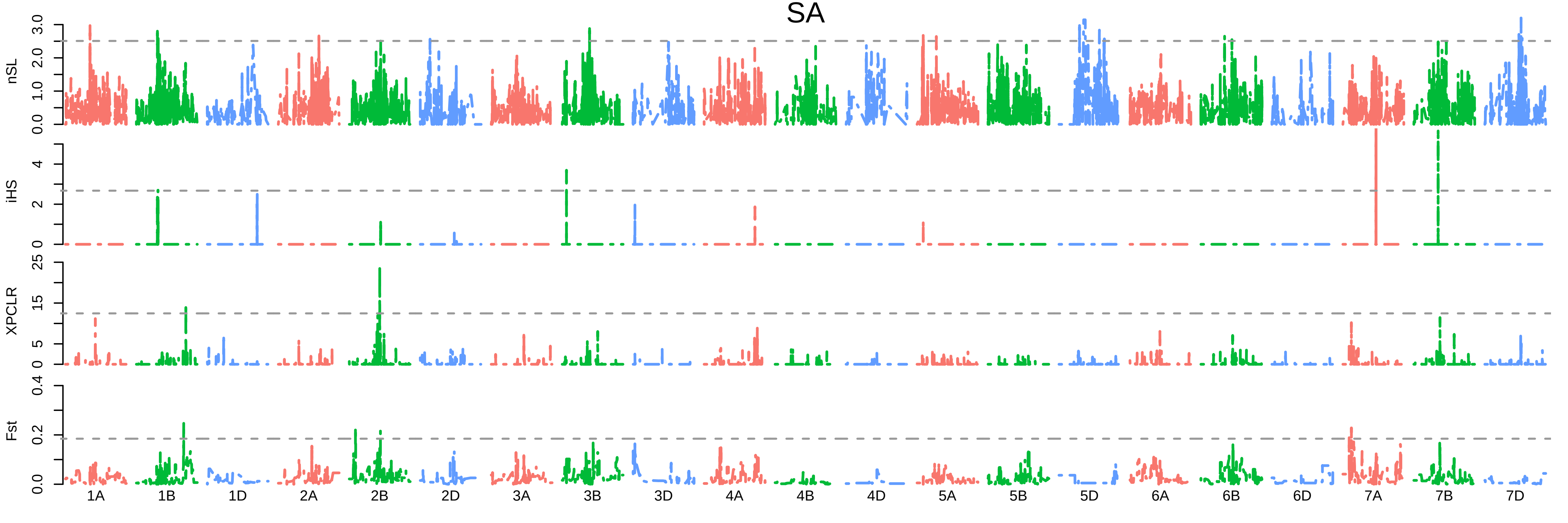
SA
nSL
iHS
XPCLR
Fst
0.0
1.0
2.0
3.0
0
2
4
0
5
15
25
0.0
0.2
0.4
1A
1B
1D
2A
2B
2D
3A
3B
3D
4A
4B
4D
5A
5B
5D
6A
6B
6D
7A
7B
7D

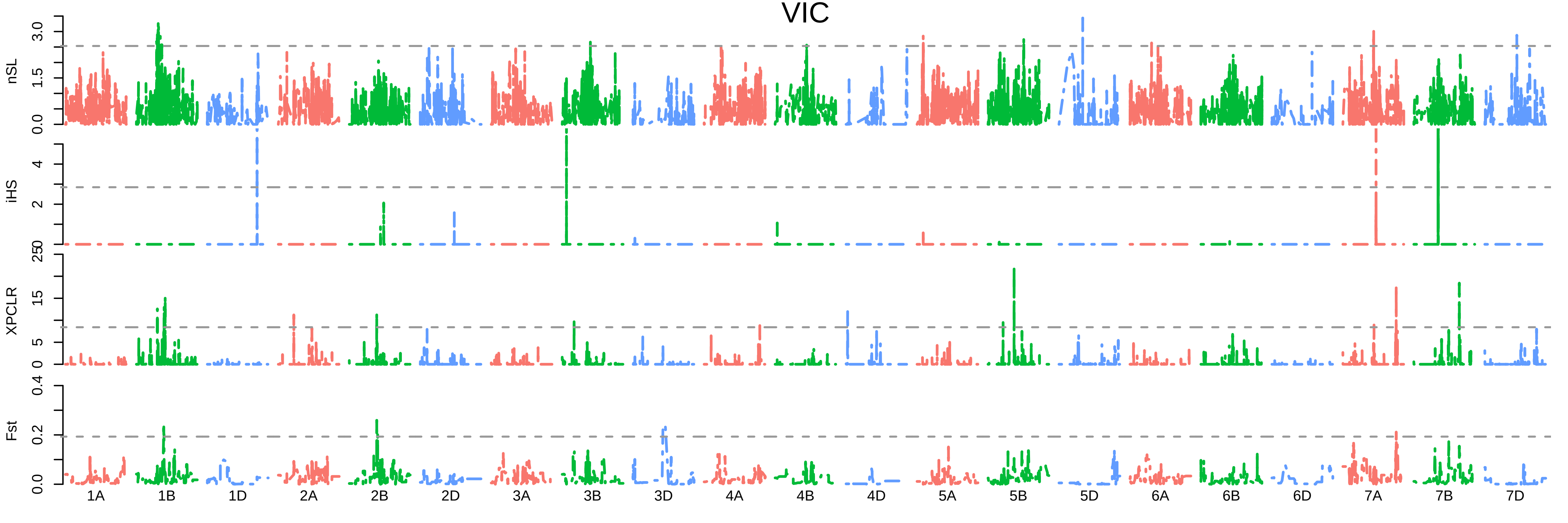

VIC
nSL
iHS
XPCLR
Fst
1A
1B
1D
2A
2B
2D
3A
3B
3D
4A
4B
4D
5A
5B
5D
6A
6B
6D
7A
7B
7D

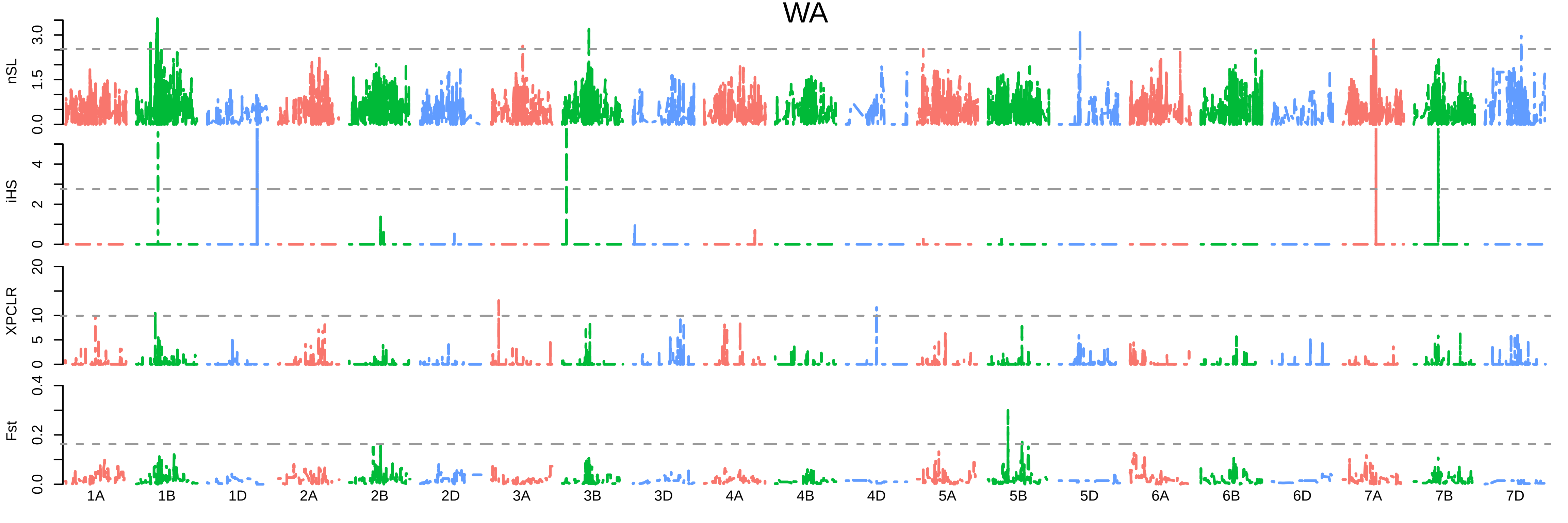
WA
nSL
0.0
1.5
3.0
iHS
0
2
4
XPCLR
0
5
10
20
Fst
0.0
0.2
0.4
1A
1B
1D
2A
2B
2D
3A
3B
3D
4A
4B
4D
5A
5B
5D
6A
6B
6D
7A
7B
7D
